# Surface or Bulk? Mechanistic Insights into Ni^2+^-Doped Brookite TiO_2_ Photocatalysts

**DOI:** 10.1021/acsnanoscienceau.5c00087

**Published:** 2025-07-30

**Authors:** Luke T. Coward, Thu T. M. Chu, Xiaotong Li, Pin Lyu, Oksana Love

**Affiliations:** † Department of Chemistry and Biochemistry, University of North Carolina Asheville, 1 University Heights, Asheville, North Carolina 28804, United States; ‡ Department of Chemistry and Organic and Carbon Electronics Laboratories (ORaCEL), North Carolina State University, Raleigh, North Carolina 27695, United States

**Keywords:** photocatalysis mechanism, transition-metal-ion doping, brookite TiO_2_, doping distribution, chemical kinetics

## Abstract

Solar energy, as an alternative source to catalyze chemical reactions, has been rapidly utilized and developed over the past few decades, particularly with TiO_2_-based semiconductor photocatalysts. Regulating the carrier dynamics under photoexcitation and controlling the interfacial reaction kinetics have been emphasized as fundamental approaches to increase the quantum yield of photocatalytic systems. Transition-metal-ion doping is a promising strategy to address these issues, although the precise roles and optimal spatial distribution of dopants remain unclear. In this systematic study, we designed surface-only, bulk-only, and surface-bulk-doped brookite TiO_2_ nanoparticles using Ni^2+^ as dopants and evaluated the photocatalytic performance of these doped samples based on the apparent reaction rate constants. It is demonstrated that the crystal structure, morphology, and surface composition did not change significantly after doping, and the observed enhancement in photocatalysis can be correlated to the doping positions. Continuous doping from the bulk to surface, forming the trap-to-transfer centers to mediate interfacial electron transfer, proves to be the most effective pathway. This proof-of-concept work offers a unique perspective on the transition-metal-ion-induced photocatalysis mechanism of brookite TiO_2_ nanoparticles and will help us design more efficient photocatalytic systems.

## Graphical abstract

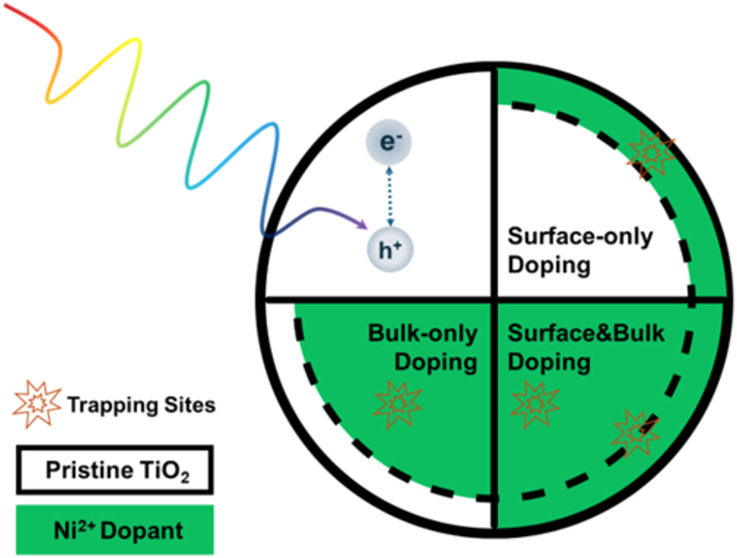

## Introduction

Semiconductor photocatalysts, particularly TiO_2_-based materials, have been developed for various chemical reactions since the initial report of rutile TiO_2_ as a photoelectrocatalyst for water splitting in 1972.^1–5^ Intrinsic TiO_2_, which consists of three common crystal phases, anatase, rutile, and brookite, has a band gap that ranges from 3.0 to 3.3 eV.^6–8^ This range permits photoabsorption only below 410 nm, primarily within the ultraviolet region, accounting for approximately 5% of the solar energy distribution.^9^ Various strategies have been proposed to enhance the visible-light response of TiO_2_-based photocatalysts,^10–12^ including dye sensitization,^13–15^ noble-metal cocatalysts,^16–18^ heterojunction structuring,^19–21^ metal/nonmetal doping,^22–24^ and defect engineering.^25–27^ Among these strategies, transition-metal-ion doping stands out as one of the earliest and most enduring methods,^28–31^ due to its versatility and flexibility in fundamentally altering electronic structure, which heavily relies on structure–property relationships. Additionally, using earth-abundant transition metals is more cost-effective and sustainable compared to noble metals or organic dyes.^32–35^

Historically, various mechanistic interpretations of metallic-ion dopants in TiO_2_, informed by energetics, optical response, and related photophysics, have been presented and were recently summarized in this perspective article by Khan and co-workers.^36^ Briefly, three representative mechanisms have been proposed: (1) the dopants can form intermediate states (or an extra absorption band if doped at a high percentage), which reduce the effective band gap and expand light absorption into the visible range;^31,37–39^ (2) the dopant redox pair can serve as a mediator, tuning the redox potential of photogenerated radicals to match the desired redox reaction pathway;^30,40–42^ and (3) the dopants can trap photogenerated carriers, increasing their lifetime by reducing the recombination rate and thus improving quantum efficiency.^29,43–45^ It is noteworthy that most of the work involves anatase and rutile TiO_2_ nanoparticles, while brookite TiO_2_ is still underexplored. More importantly, the exact role of transition-metal-ion dopants remains unclear, particularly in light of the controversial literature regarding either enhanced or deteriorated photocatalytic activities.^29,46–48^ This may be due to the specific synthetic methods, the actual photolysis, and the broad range of chemical reactions examined across a wide spectrum of wavelengths, which necessitated a more systematic approach to address this issue.

Fundamentally, upon photoexcitation with a photon energy greater than the band gap, the electrons in the semiconductor are promoted from the occupied valence band (VB) to the unoccupied conduction band (CB), generating excited electron–hole (e^–^/h^+^) pairs. The photogenerated charge carriers can migrate to the surface to participate in redox reactions, either lowering the activation barrier or increasing the reaction rate. When transition-metal ions are doped into the TiO_2_ crystal lattice, they change the carrier dynamics from intrinsic defect trapping (bulk trapping >20 ns and surface trapping at 1–20 ps) to artificial trapping sites (bulk/surface to 200 ns on average), as measured by transient absorption spectroscopy.^49^ One of the key factors influencing the final photocatalytic performance is that the dopant, whether located on the surface or within the bulk, must act as a trapping site to decrease the level of electron–hole recombination. This action enhances the lifetime of photogenerated charge carriers and facilitates interfacial charge transfer, thereby promoting chemical reactions. In fact, this is not always the case, as the trapping effect can be two sides of the same coin;^49–51^ deep trapping in the bulk may reduce the number of charge carriers reaching the surface. In contrast, shallow trapping at the surface may induce extra intraatomic d-d transitions that annihilate the charge carriers. Therefore, a more nuanced design approach is necessary to distinguish and potentially balance these two aspects.

For this study, the nickel­(II) ion (68 pm) is selected as the exemplary candidate for the doping effect due to its radius similar to that of the titanium­(IV) ion (72 pm) and a relatively mild energy level that lies between the conduction and valence bands. Doping of anatase TiO_2_ with Ni^2+^ has been reported in the literature and surface-bulk doping has been shown to be successful, whereas surface-only doping is less common. Ni^2+^ doping of brookite structure is highly under-researched, and most of the reports employed surface-bulk doping methods.^52–54^ In this work, we utilized hydrothermal method for synthesis of pristine and doped TiO_2_ and systematically designed the distribution of dopants within the crystal surface, within the bulk, or both (Scheme 1). These Ni^2+^ ions can readily replenish the Ti^4+^ or Ti^3+^ defect sites in the pristine TiO_2_ nanoparticles, achieving the desired doping status through various synthetic or post-treatment methods. This approach could limit structural distortion and morphological changes across different samples, narrowing down the only contributing factor to the dopant position. Furthermore, the photocatalytic performance of methylene blue degradation is employed as a model reaction to evaluate this contribution and correlate it with the doping effect. Finally, mechanisms of photocatalysis involving different doping locations are proposed to elucidate the exact roles of these dopants and provide unique insights into transition-metal-ion-doped photocatalysts.

**Scheme 1:**
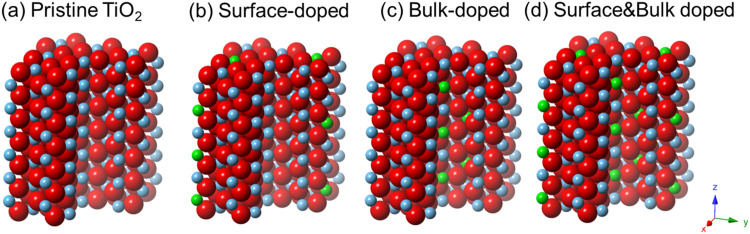
Schematic Representations of Different Doping Locations into Brookite TiO_2_: (a) Pristine (Pristine TiO_2_), (b) Surface-Doped Only (Ni^2+^-TiO_2_–Surface), (c) Bulk-Doped Only (Ni^2+^-TiO_2_–Bulk), and (d) Surface-Bulk-Doped (Ni^2+^-TiO_2_–Surface & Bulk)^a^

a Red stands for oxygen, blue for titanium, and green for nickel.

## Experimental Methods

### Chemicals and Characterizations

All chemicals and reagents were used without any purification. The morphology of the nanoparticles and elemental compositions of dopants were studied by scanning electron microscopy with energy-dispersive X-ray spectroscopy (SEM-EDS, SEM-JSM-IT700HR, 15 kV, JEOL with an EDS-Ultim Max 40 detector, Oxford Instruments). For nickel quantitative analysis, the EDS detector was calibrated with UHV-EL Reference Standards for EDS/WDS (99.994% purity of nickel in crystalline form, Ted Pella, Inc., stored in a vacuum desiccator). The high-resolution spatial distribution was confirmed by high-angle annular dark-field scanning transmission electron microscopy (HAADF-STEM) with energy-dispersive X-ray spectroscopy (EDS) (Thermo Fisher Talos F200X S/TEM operated at 200 kV). Images and EDS maps were acquired in STEM mode, using a convergence angle of ∼10.5 mrad on the HAADF detector. EDS maps were processed to subtract the background and deconvolute any overlapping peaks by using standard quantification routines in the Velox software package. Optical diffuse reflectance measurements were collected using a Shimadzu UV-3600 UV–vis NIR spectrometer in the spectral range of 200–1000 nm at room temperature (Ba_2_SO_4_ was used as a reference). To correctly determine the band gap energies of the samples, the methyl orange and the samples were diluted with Ba_2_SO_4_ in a 1:100 weight ratio and placed side by side in the sample holder.^55^ The local crystalline structure of pristine and doped TiO_2_ nanoparticles was determined by the powder X-ray diffraction pattern (Bruker D2 PHASER Benchtop XRD, Cu Kα radiation, 30 kV, 10 mA) and was processed with DIFFRAC.EVA data analysis software with the Crystallography Open Database (rev. 278581). The Rietveld refinements of the data were performed in the BGMN kernel using the open-source software Profex.^56^ The unpaired electrons in doped TiO_2_ nanoparticles (dopant ions or oxygen vacancies) were measured by electron paramagnetic resonance (EPR) spectroscopy (Bruker EMXnano Benchtop EPR system, X-band, microwave frequency of 9.61 GHz, and power attenuation of 4.00 dB). The samples were ground using mortar and pestle before being loaded into a 4 mm (outer diameter) EPR quartz sample tube. The overall atomic percentage of Ni dopants across the sample was measured by inductively coupled plasma optical emission spectroscopy (ICP-OES, Agilent 5110 ICP-OES). The instrument parameters are radio frequency power of 1.2 kW, plasma flow of 15.0 L/min, auxiliary flow of 1.50 L/min, nebulizer flow of 0.75 L/min, sample uptake delay of 15 s, instrument stabilization delay of 15 s, replicate read time of 2 s, and replicates of 3 times. The sample (approximately 0.05 g) was digested with a mixture of concentrated nitric acid (HNO_3_) and hydrofluoric acid (HF), and then diluted 10 times for the ICP-OES test. The surface composition and oxidation states were determined by X-ray photoelectron spectroscopy (XPS, Kratos Analytical Axis Ultra system with monochromatic Al–Kα X-ray source operated at 150 W), in which the contamination carbon (C–C, at 284.9 eV) was set as the reference to calibrate the binding energy of other elements. Charge neutralization was applied when necessary to prevent sample charging. The surface composition with functional group identifications was determined by Attenuated Total Reflectance Fourier Transform Infrared spectroscopy (ATR-FTIR, SHIMADZU IRSpirit-X).

### Synthesis of Pristine Brookite TiO_2_ Nanoparticles

The synthesis procedure was modified from the hydrothermal method reported by Li and co-workers.^57^ In brief, 0.6 g of titanium­(IV) oxysulfate-sulfuric acid hydrate (TiOSO_4_·*x*H_2_O + *x*H_2_SO_4_) was added to 12.5 mL of deionized water (DI H_2_O) and stirred at 700 rpm and room temperature for 1 h, until the solution became clear and all precursors were fully dissolved. Then, 25 mL of 0.5 M sodium hydroxide (NaOH) solution was added to adjust the pH to 12.5, and the formed white sol–gel precipitate was stirred for an additional 24 h. The sol–gel solution was then purified to remove any leftover precursors by centrifugation at 5000 rpm for 5 min (5 times). The final pH of the sol–gel solution was adjusted to 12.5 with 6 M NaOH­(aq) before being sealed into a PPL-lined hydrothermal autoclave and heated to 220 °C for 24 h. After being cooled to room temperature, the pristine brookite TiO_2_ nanoparticles were filtered and washed with DI H_2_O and ethanol. The dry powder samples were collected and stored in the refrigerator for further characterization and photocatalysis.

### Synthesis of Surface-Only Ni^2+^-Doped TiO_2_ Nanoparticles

The surface-only-doped samples, Ni^2+^-TiO_2_–surface, were synthesized from the pristine brookite TiO_2_ nanoparticles. 0.135 g of pristine brookite TiO_2_ nanoparticles was mixed with 16 mg nickel­(II) chloride hexahydrate (NiCl_2_·6H_2_O) in 10 mL of DI H_2_O under constant stirring, and the mixture was sonicated for 5 min and then heated up to 120 °C until all water was evaporated. The weight percentage of Ni^2+^ dopant to Ti was prepared to be 4% in the mixture solution, which was twice as high as the surface-bulk-doped sample below due to the low doping efficiency of this method. Then, the mixture was heated to 350 °C for 3 h to dry the particles. The final surface-only Ni^2+^-doped TiO_2_ nanoparticles were washed with DI H_2_O and ethanol.

### Synthesis of Surface-Bulk Ni^2+^-Doped TiO_2_ Nanoparticles

The synthesis procedure for surface-bulk samples, Ni^2+^-TiO_2_–Surface-Bulk, was followed similar to the pristine brookite TiO_2_ nanoparticles above; except in the first step, 8 mg of NiCl_2_·6H_2_O was added into the titanium­(IV) oxysulfate-sulfuric acid hydrate solution to serve as the dopant agents. The weight percentage of Ni^2+^ dopant to Ti was prepared to be 2% in the mixture solution.

### Synthesis of Bulk-Only Ni^2+^-Doped TiO_2_ Nanoparticles

The bulk-only-doped samples, Ni^2+^-TiO_2_–bulk, were synthesized based on the surface-bulk Ni^2+^-doped TiO_2_ nanoparticles procedure. 0.2 g of surface-bulk Ni^2+^-doped TiO_2_ nanoparticles was added to 10 mL of 3 M hydrochloric acid (HCl) solution and stirred under 700 rpm for 3 h to remove the surface-doped Ni^2+^ ions. The mixture solution was then filtered and washed with DI H_2_O and ethanol. Acid-washing the surface of the nanoparticles resulted in a greenish solution after filtration, indicating the removal of surface-bonded Ni^2+^.

### Photocatalytic Performance of Pristine and Doped TiO_2_ Nanoparticles

The methylene blue (MB) dye degradation served as a model reaction to compare the photocatalytic performance of these nanoparticles. In a typical procedure, 6 mg of nanoparticles (0.0025 mol/L TiO_2_) was mixed with 30 mL of 10 ppm MB solution (3.1 × 10^–5^ mol/L) in a quartz reaction tube. The solution mixtures were stirred and sonicated to ensure complete mixing before the reaction. The dark adsorption–desorption equilibrium was implemented to account for any differences in the adsorption capacity for the different nanoparticles. Then, two different light sources were introduced into the system to perform the photocatalytic reactions. The visible-light reaction setup consisted of a photoreactor (Rayonet RPR-100 Photochemical Reactor) with six visible lamps (center wavelength of 575 nm, approximately 91 mW/cm^2^ at the center) surrounding the quartz tube. The full-wavelength setup utilized a Mercury Arc Lamp as the light source (Oriel 68111 Hg Arc Lamp Power Supply, 6283NS Mercury lamp, 200 W). All photoreactions were conducted under cooling fans to maintain a stable room temperature. The degradation process was monitored by taking 3 mL solution samples every 5 min and centrifuging at 14,000 rpm to remove the nanoparticles. The absorbance of the MB dye was measured by a UV–vis spectrometer (Shimadzu UV–vis 1800). The dye concentrations were calculated with a calibration curve ranging from 0.5 to 10 ppm with a limit of detection of 0.3 ppm and a limit of quantification of 0.9 ppm. The control experiment to confirm the contribution of superoxide radicals was performed by purging Ar into the reaction tube before the reaction, followed by the same procedure as that above. The control experiment to confirm the contribution of hydroxyl radicals was performed by adding 1 mL of methanol into the reaction tube before the reaction, with the same procedure subsequently carried out.

## Results and Discussion

The pristine brookite TiO_2_ nanoparticles were synthesized using a modified hydrothermal method,^57^ achieving high crystallinity and phase purity. The surface-bulk-doped samples were prepared via the same method, where Ni^2+^ ions were introduced into the precursor solution. The bulk-only samples were created from the surface-bulk-doped samples by acid-washing the surface Ni^2+^, producing a slightly green solution during the washing and rinsing process, indicating the removal of surface-bonded Ni^2+^. The surface-only samples were prepared from pristine nanoparticles by doubling the dopant concentration. This mixture underwent an evaporation and calcination process to anchor the Ni^2+^ on the surface more effectively. The higher percentage of dopants used compensated for the inefficiency of the surface-replacement method and the dynamic equilibrium of Ni^2+^ at the surface and in solution. The color of the final powder samples gradually evolved from white to light yellow and then to dark yellow across pristine, surface-only, bulk-only, and surface-bulk-doped samples, as shown in Figure S1. This evolution indicates successful doping and reflects the relative strength of the doping effect on light absorption. The darker color suggests that more dopants have either substituted into the crystal lattice or anchored on the surface, leading to a reduced band gap, as demonstrated in Figure S2. The band gap measurement was modified from the Kubelka–Munk function, using methyl orange as a baseline, to account for surface modification and doping contributions to light absorption.^55^ The surface-bulk-doped and surface-only samples exhibited a noticeable band gap shift to 2.9 eV from 3.1 eV in the pristine samples, while bulk-only samples showed shifts of about 0.1 eV (Figure S2). As mentioned in the introduction, the relatively low doping percentage and small changes in the band gap suggest that the individual trapping sites formed by the dopants across different samples exclude the contribution of extra visible-light-band-induced photocatalysis mechanisms. Still, it is of our future interest to increase the dopant percentage to identify this threshold change and to explore these different photoexcitation and reaction pathways.

The crystal structure and morphology of the synthesized nanoparticles are critical for carrier generation and migration under photoexcitation, particularly when extrinsic dopants are introduced into the lattice or onto the surface. As shown in Figure 1, there are almost no changes in the overall structure of all Ni^2+^-doped samples compared to the pristine brookite TiO_2_ nanoparticles, in which the pristine sample was compared to the PDF standard card to confirm that there was no phase impurity (Figure S3). The Rietveld refinement analysis of the pristine and doped samples in Figure S4 demonstrated a relatively good match between the calculated spectra and the standard brookite TiO_2_ spectrum with an acceptable goodness of fit of less than 9% for all samples. Additionally, the lattice parameters showed very limited changes across the pristine and doped samples, indicating the good dispersion of Ni^2+^ and intact crystal structure after doping. All of the samples are highly crystalline, as shown in Table S1, indicating the integrity of the overall structure of all doped samples. No distinct Ni peaks are observed in the diffraction patterns of all doped samples, indicating a relatively well-dispersed Ni^2+^ distribution in the lattice or on the surface. In addition, no NiO_2_ and Ni_2_O_3_ peaks were observed, pointing toward the successful doping discussed in the later sections. The enlarged section in Figure 1b is the signature doublet peak of the brookite (210) and (111) crystal planes. There are no significant changes in the peak positions and interplanar *d*-spacing for pristine and surface-only and bulk-only-doped samples (Table S1). Among all samples, only the surface-bulk samples exhibit a discernible shift, characterized by the doublet peaks moving to slightly lower angles compared to the pristine TiO_2_. This could be due to a relatively higher substitutional doping percentage, causing lattice expansion. This observation is not surprising since the final dopant concentration for all samples was very low so as not to distort the final crystal structure. Photoexcitation is primarily a bulk phenomenon, relying on the overall band structure and density of states of the conduction and valence bands. As mentioned above, the overall crystal structure is quite similar among doped and pristine samples, and there should not be much difference in terms of the photoexcitation pathway.

**Figure 1:**
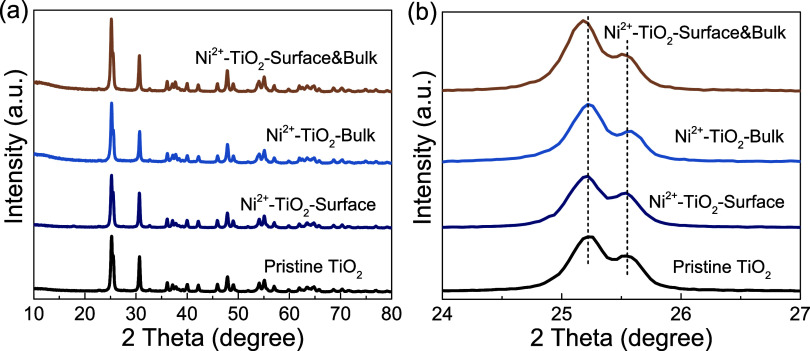
(a) X-ray diffraction (XRD) patterns of pristine brookite TiO_2_, surface-only, bulk-only, and surface-bulk-doped TiO_2_ with Ni^2+^. (b) Enlarged part between 24 and 27 degrees of (a).

After photoexcitation, electrons are promoted from the valence band to the conduction band, where most of the electron–hole pairs recombine, resulting in the re-emission of a photon or the dissipation of phonon energy into the lattice heat. A small percentage of the photogenerated carriers migrate across the surface of the photocatalysts to participate in interfacial redox reactions. Therefore, it is critical to assess the changes in the morphology and the presence of surface functional groups following the doping process. As shown in Figure 2a–c, the pristine brookite TiO_2_ nanoparticles tend to grow into spindle-like shapes due to the relatively small surface formation energy of the (001) planes compared to the (100) planes.^58^ As suggested in the original synthesis report,^57^ these small particles undergo a further Ostwald ripening process, growing and welding together to form an assembly-like microstructure, ultimately resulting in a stacked flower-like structure, as observed in our SEM as well. As the surface-only-doped samples derive directly from the pristine samples, there are no changes in morphology, as shown in Figure 2d–2f.

**Figure 2:**
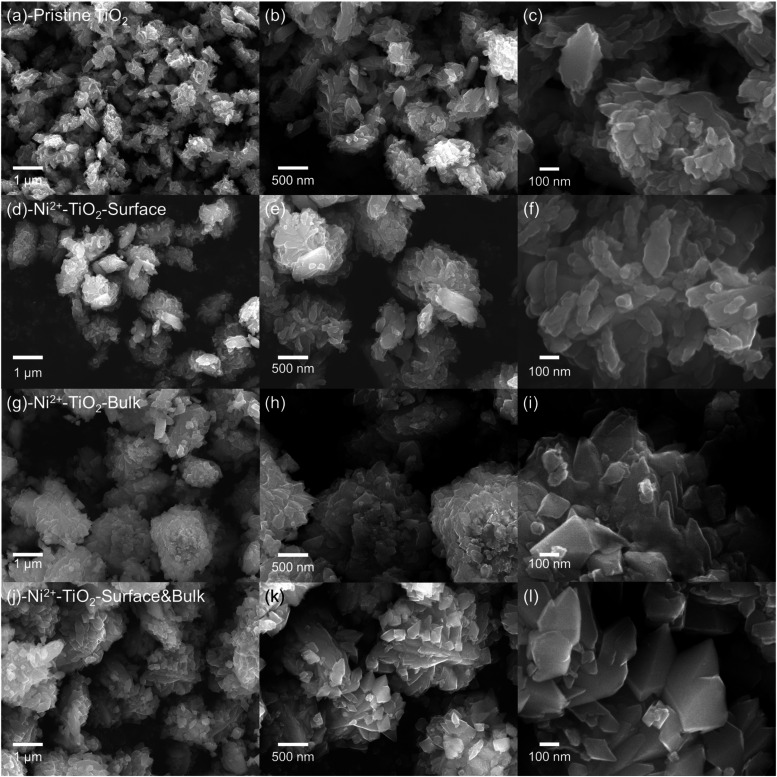
SEM images of (a–c) pristine brookite TiO_2_ nanoparticles, (d–f) surface-only Ni^2+^-doped, (g–i) bulk-only Ni^2+^-doped, and (j–l) surface-bulk Ni^2+^-doped TiO_2_ nanoparticles.

For the surface-bulk-doped samples, since the Ni^2+^ ions are directly involved in the precursor solutions and the entire crystallization process, it is not surprising that there may be some minor morphological changes. As shown in Figure 2j–2l, clearer plane boundaries are observed for individual elongated octahedral structures, which suggests that the doped ions promote the formation of thermodynamically stable Wulff constructions with more exposed uncoordinated Ti_5c_ {210} facets.^58–60^ These facets enhance the adsorption of reactants and subsequent photocatalytic interfacial electron transfer, as discussed in the section below. It is noted that a higher percentage of doping ions distorts the crystal structure by breaking the local symmetry of the TiO_6_ octahedron building units and leads to the formation of undesired shapes or phase impurities,^61^ which is not observed in our synthetic protocol due to low doping amount.

Furthermore, the bulk-only-doped sample is obtained from the surface-bulk-doped samples through acid-washing. The overall structure remains unchanged, as shown in Figure 2g–2i, although the sharp edges and corners are now more truncated and rounded. However, it is noteworthy that all doped samples remain highly crystalline, as discussed above, and there are no separate peaks for nickel or nickel oxide in XRD and XPS analysis. The minor deviation in morphology is almost negligible in all doped samples compared to the pristine samples, which should not be the dominant factor when discussing the photocatalytic performance in the later section.

Since the reaction predominantly occurs on the catalyst’s surface, FTIR is employed to examine the surface functional groups of all samples. As shown in Figure S5, the brookite TiO_2_ nanoparticles are largely hydrophobic on the surface, containing few hydroxyl groups, but exhibiting a significant peak around ∼2369 cm^–1^ that corresponds to surface-adsorbed CO_2_.^62,63^ It is possible that defects, either Ti^3+^ or oxygen vacancies, serve as active sites for the adsorption of atmospheric carbon dioxide,^64,65^ as confirmed by the XPS C 1s results discussed later. After doping, the only significant change occurs in the bulk-only samples (postacid washing), where more hydroxyl groups emerge; however, the defect sites remain, which correlates with the later XPS results. Thus, the overall crystal structure, morphology, and surface status after Ni^2+^ doping have shown very limited changes, which rules out other contributions to the correlation with the Ni^2+^ dopant trapping effect on the final photocatalytic performance.

Moving forward, quantifying the actual doping percentage and distinguishing between the surface and bulk doping distributions are key factors for deciphering the photocatalysis mechanism. First of all, SEM-EDS mapping and elemental composition, calibrated with a high-purity nickel standard, confirmed the existence and well-dispersed distribution of doped Ni^2+^ ions in all doped samples, as shown in Figures S6–S8. Additionally, high-angle annular dark-field scanning transmission electron microscopy (HAADF-STEM) with EDS elemental mapping confirmed the spatial distribution of dopants as shown in Figure 3. The surface-only-doped sample demonstrated a relatively rich distribution of Ni at the edge of the particle, while the bulk-only-doped sample showed a rich distribution of Ni in the bulk and a deficient distribution at the edges. For the surface-bulk-doped sample, the distribution of Ni was relatively uniform across the sample.

**Figure 3:**
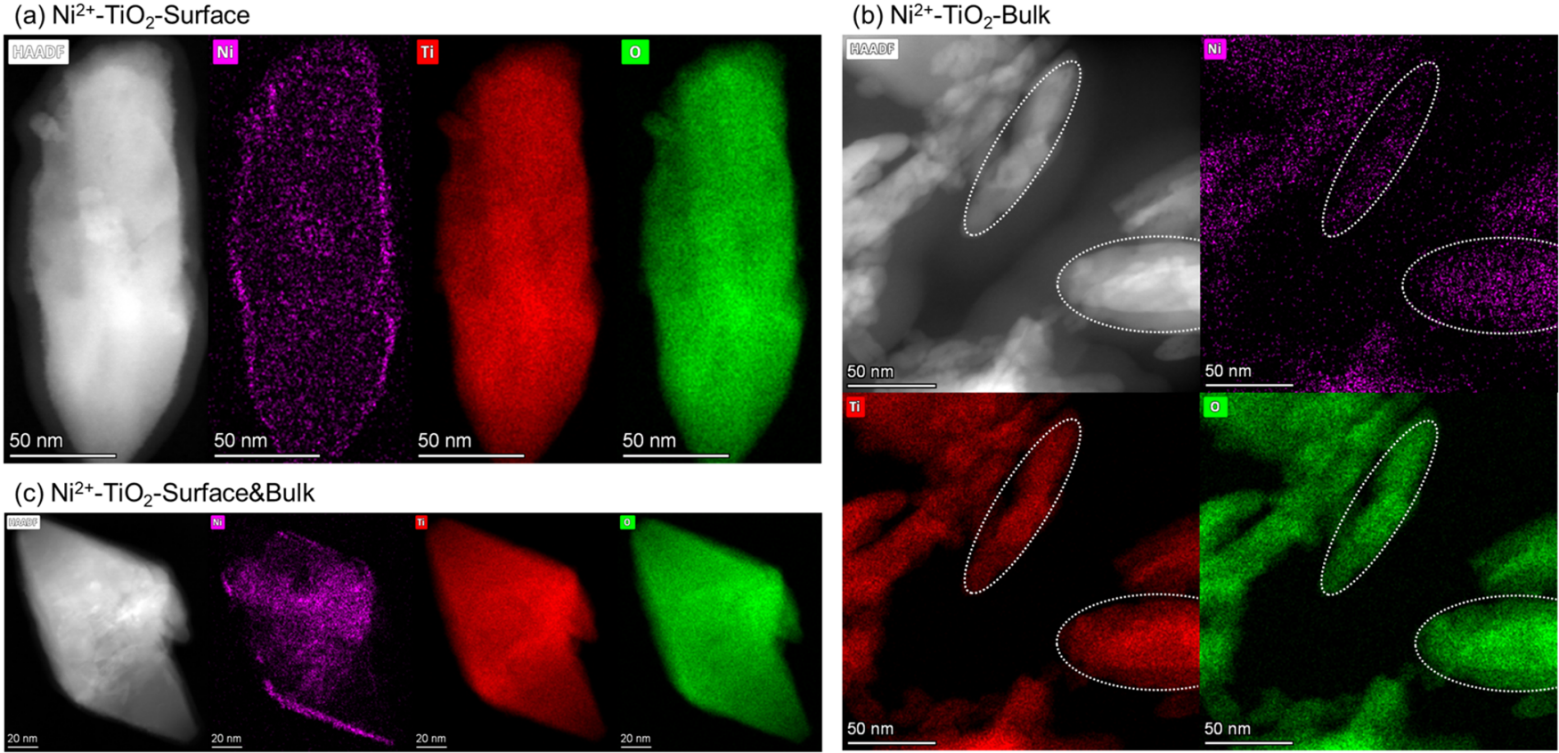
HAADF-STEM images and elemental mapping of Ni, Ti, and O across the particles of (a) surface-only Ni^2+^-doped, (b) bulk-only Ni^2+^-doped, and (c) surface-bulk Ni^2+^-doped brookite TiO_2_ nanoparticles. The white dashed lines in panel (b) were used to guide the comparison of the distributions of different elements.

Electron paramagnetic resonance (EPR) spectroscopy was used to qualitatively assess the defect concentrations in pristine and doped samples. The intrinsic defect-rich brookite crystal structure offers numerous active sites for substituting the Ti center with Ni ions. The broad peak and high intensity of free electrons at the defect sites could override the signal for Ni^2+^. The Ni^2+^ signal of *g* = 2.07, indicating the substitutional doping,^66^ can be observed in the sample after exposure to air for an extended period of time, as shown in Figure S9. It is within our current progress to further track the depletion of defects over time and the migration of dopants inside the lattice under a controlled laboratory environment. Nonetheless, the spin concentration of free electrons can still be correlated to the defect concentration and Ni^2+^ dopants in the overall structure of the nanoparticles.

The pristine TiO_2_ sample was prepared through high-temperature hydrothermal synthesis under strong alkaline conditions (pH = 12.5), where the excess OH^–^ could potentially occupy the O^2–^ sites on the surface growth units and stabilize these intrinsic defect sites.^67^ As shown in Figure 4a, the pristine TiO_2_ exhibits a very intense and broad resonance peak with a *g*-factor of 2.00 in the room-temperature solid-state EPR spectrum, which matches the resonance signal of free electrons (*S* = 1/2) located on the defect sites. The asymmetric feature of the peak suggests the existence of at least two different sources of free electrons in different local environments, in which the oxygen vacancies and Ti^3+^ defects could be the main contribution.^68^ The low-valent Ti^3+^ defects and oxygen vacancies are later confirmed in the high-resolution XPS spectra (Figure 5a,5b). These unique structural defects provide space and lower energy barriers for the substitutional doping of Ni^2+^ ions into the lattice or on the surface. For the surface-only sample in Figure 4b, a much lower intensity is observed and more localized information about the Ni^2+^ dopants is indicated in the rather noisy signal at a lower magnetic field. The broad peak with a *g*-factor of 2.12 suggests substitutional doping of Ni^2+^ onto the surface,^66^ and the more intense peak with a *g*-factor of 2.00 indicates some bulk defects remaining after the surface-replacement method. The feature of this substitution peak is also observed in the EPR spectrum of the surface-bulk-doped sample with less defects in Figure S9. Furthermore, the surface-bulk-doped sample in Figure 4d shows a significantly narrower resonance peak, suggesting more delocalized free electrons surrounding the Ti or Ni centers in the lattice structure.^69^ After acid-washing, the bulk-only sample in Figure 4c demonstrates a stronger resonance peak compared with the surface-bulk-doped sample, indicating a higher defect concentration. Overall, these results estimate the relative concentration of defects (localized free electrons) and Ni^2+^ dopants among pristine and doped samples, offering valuable insights for the subsequent discussion of surface and bulk distinctions.

**Figure 4:**
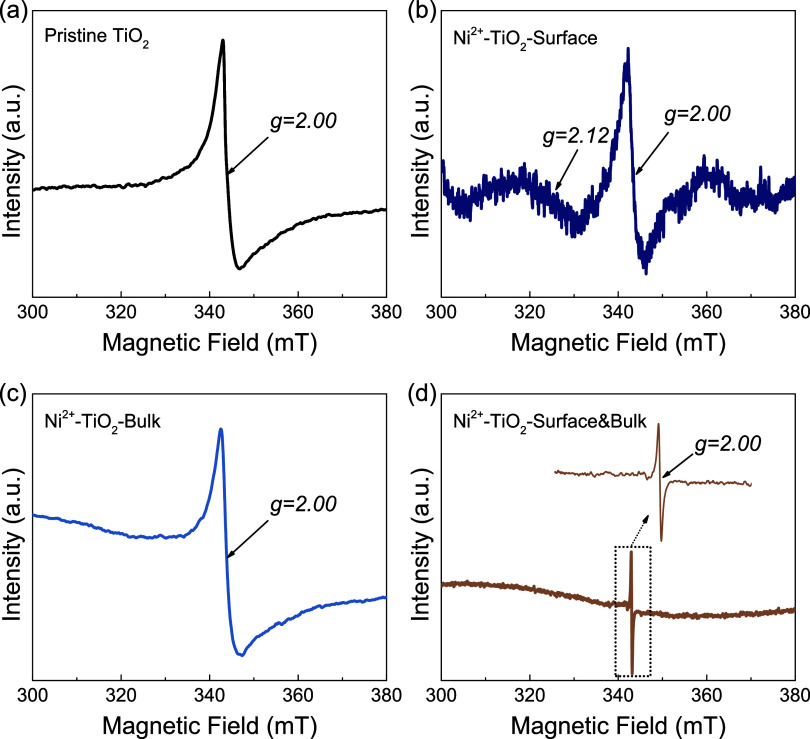
Solid-state EPR spectra of (a) pristine brookite TiO_2_ nanoparticles, (b) surface-only Ni^2+^-doped, (c) bulk-only Ni^2+^-doped, and (d) surface-bulk Ni^2+^-doped brookite TiO_2_ nanoparticles. The top right inserted image in panel (d) is the enlarged part of the surface-bulk-doped sample between 335 and 350 mT.

**Figure 5:**
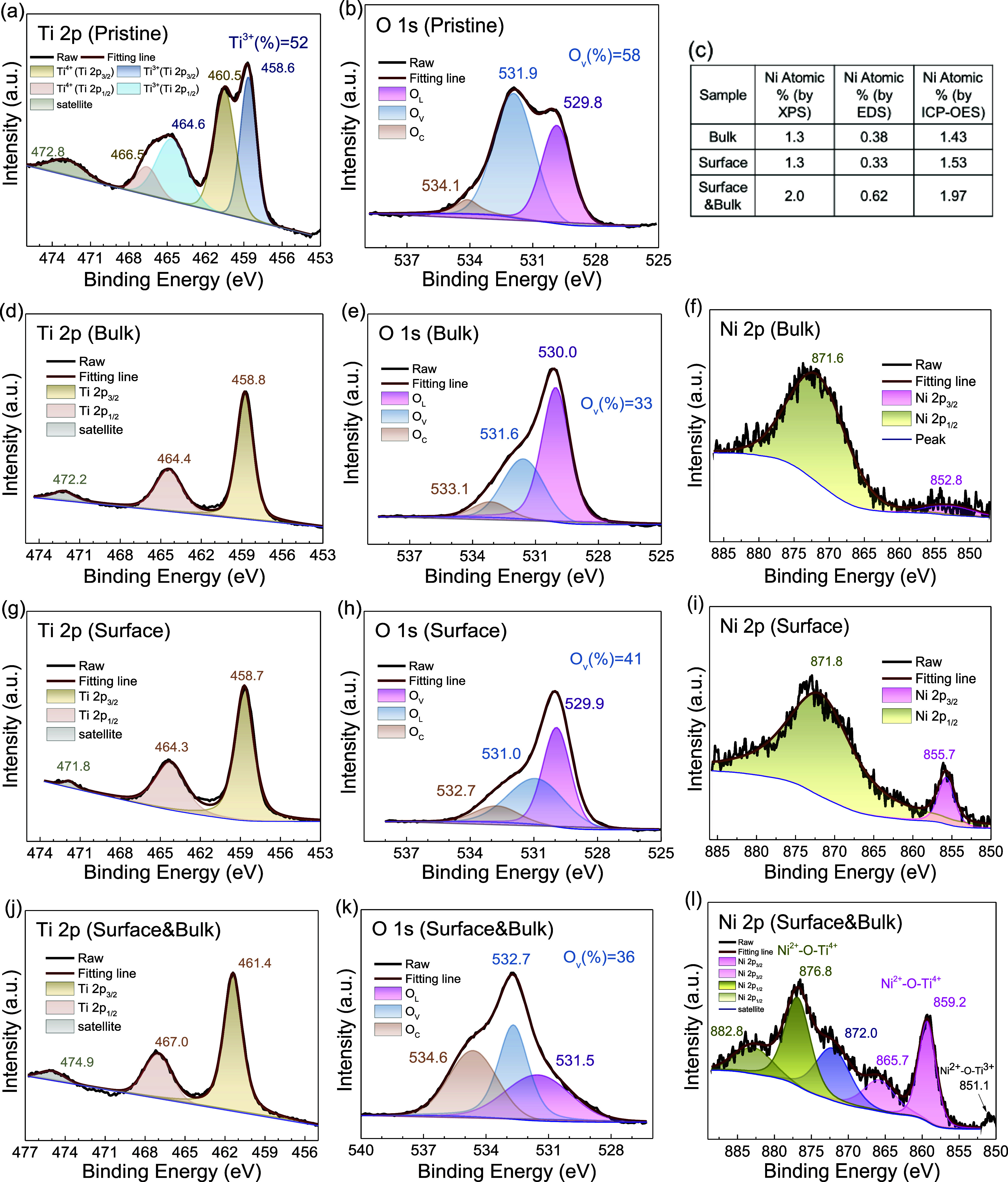
Spectroscopic evidence of doping states into brookite TiO_2_ nanoparticles. High-resolution XPS spectra of Ti 2p, O 1s, and Ni 2p in (a, b) pristine brookite TiO_2_ nanoparticles, (d–f) bulk-only-doped, (g–i) surface-only-doped, and (j–l) surface-bulk-doped brookite TiO_2_ nanoparticles. The atomic percentage of Ni dopants was calculated from the XPS full-range survey listed in panel (c) and compared with the ratio from the SEM-EDS elemental analysis. O_L_ represents lattice oxygen, O_v_ for oxygen vacancies/defects and O_c_ for chemisorbed oxygen species.

As follows, the solid-state EPR provides information about the overall defect concentration throughout the sample, while the XPS high-resolution spectrum is more sensitive to the surface or subsurface, extending to a few nanometers. As shown in Figure 5a, both Ti 2p_3/2_ and Ti 2p_1/2_ signals of Ti^4+^ exhibit shoulder peaks at lower binding energy (of Ti^3+^), while the Ti 2p_3/2_ signal of Ti^3+^ is more evident with a separated peak. This result confirms the existence of Ti^3+^ as one of the defects mentioned above in the EPR spectrum in Figure 4a. Furthermore, based on the peak deconvolution results in Figure 5a,5b, pristine brookite TiO_2_ nanoparticles exhibit a high percentage of Ti^3+^ (∼52%) and oxygen vacancies (∼58%) on their surface, which aligns with the EPR observation mentioned above. The defect sites are predominantly electron-rich, making them prone to the adsorption of carboxylic acids or carbon oxides from ambient air,^70–72^ as shown in the XPS C 1s high-resolution spectrum in Figure S10. This aligns with our earlier discussion of FTIR analysis. These surface-adsorbed species are also evident in the high-resolution O 1s spectrum, which shows a distinct shoulder peak at higher binding energy, attributed to chemisorbed oxygen species. Introducing ions that can potentially fill or replace these defect sites will significantly alter the surface structure, also leading to the possibility of the Ti–O–Ni bond formation. More ultrahigh vacuum studies need to be pursued to monitor surface changes *in situ* to reconstruct the overall doping process,^73–75^ which is beyond the scope of this work.

After doping, the most noticeable change in the XPS spectra is the absence of a shoulder peak and the narrowing of Ti 2p peaks in Figure 5d,5g,5j, indicating that the Ni^2+^ ions replace the Ti^3+^ defect sites on the surface and possibly follow a similar process in the bulk.^76^ More specifically, since the bulk-only sample comes from acid-washing of the surface-bulk-doped sample, the overall spectrum features of Ti 2p resemble each other, while the O 1s and Ni 2p spectra differ significantly. It is noted that the XPS penetration depth is up to a few nanometers, which means it detects elemental signals for both the surface and subsurface. The acid-washing can only remove the surface but not the subsurface substituted Ni^2+^, which explains the similar Ti 2p spectra. However, the indirect evidence of a decreased amount of Ni^2+^ on the surface/subsurface (2.0–1.3% in Figure 5c) from the surface-bulk-doped sample to the bulk-only sample suggests successful surface removal.

Meanwhile, the oxygen vacancies/defects decreased to a lower percentage after the doping process, aligned with the observation in the EPR results above. The O 1s signal of chemisorbed oxygen species (O_c_) was observed in all doped samples, with the surface-bulk-doped sample showing a much higher percentage than the others (Figure 5). It is possible that the delocalized free electrons, as observed in the above EPR spectrum, provide more active sites for the adsorption of atmospheric carbon-involved species (as observed in FTIR, Figure S5). The Ni^2+^ ions are observed in all doped samples, where the surface-bulk-doped samples show a much clearer orbital splitting of Ni 2p, indicating that Ni^2+^ exists within the sample and is doped into the TiO_2_ lattice. The significant binding energy shift in the Ti 2p and Ni 2p core levels in surface-bulk-doped sample (Figure 5j–5l) compared to surface-only (Figure 5g–5i) or bulk-only samples (Figure 5d–5f) was possibly coming from the combination effect of chemical shift (oxidation state change) and electronic shift (relative Fermi level change between sample and analyzer).^77^ When the dopant concentration is high enough to alter the Fermi level (as observed in our surface-bulk-doped samples), the electronic shift becomes dominant and will affect all core levels of constituent elements (as observed in O 1s as well). It is noteworthy this electronic source shift is less known and often neglected, which deserves more thorough work on theoretical modeling and experimental measurements.^77^ Furthermore, even considering this large electronic shift, the Ni 2p_3/2_ binding energy value in the surface-bulk-doped sample in Figure 5l (859.2 eV) is still much higher than the typical range of those in NiO (around 856.0 eV for Ni 2p_3/2_) or Ni­(OH)_2_ (856.8 eV for Ni 2p_3/2_).^78,79^ Notably, the Ni 2p_3/2_ peak in NiO typically exhibits a doublet, which is absent in our spectrum. Similarly, Ni­(OH)_2_ is expected to show a distinct OH^–^ peak in the O 1s region, which is also not present in our system.^78,79^ Moreover, the formation of these oxides or hydroxides would be expected to significantly alter the TiO_2_ lattice structure; however, our previous XRD analysis shows no such changes. As so, the most probable configuration of the Ni^2+^ doped into the TiO_2_ is to substitute the Ti^4+^ or Ti^3+^ sites to form Ni^2+^–O–Ti^4+^ or Ni^2+^–O–Ti^3+^ bonding. The former bond is observed in the core peaks of Ni 2p_3/2_ and Ni 2p_1/2_ with clear orbital splitting. The latter is observed in a lower binding energy, for example, in Figure 5f, at 851.1 eV. Since the Ti^3+^ is already mostly replaced by the Ni^2+^, this feature is not obvious in the Ti 2p spectrum in Figure 5j. Another note about the satellite peak in Figure 5l at 872.0 eV, which is more than 10 eV compared to the core Ni 2p_3/2_ peak (at 859.2 eV), is possibly coming from removing the core electron, leading to the finite overlap of the frozen ground state with the unscreened final state of mainly 3d^8^ character.^80^

The estimation of the atomic percentage from the full-range XPS survey aligns with the trend observed in the EDS analysis mentioned above, where the surface-only and bulk-only samples exhibit similar percentages on the nanoparticle surface and throughout the structure, as shown in Figure 5c. This similarity is crucial for evaluating the photocatalytic performance discussed below. The surface-bulk-doped sample is observed with nearly double the doping percentage in both surface/subsurface XPS analysis (2.0–1.3%) and overall EDS analysis (0.6–0.3%) compared to surface-only and bulk-only samples. Additionally, ICP-OES measurements of the digested samples gave a similar trend of these samples, where the surface-bulk sample was 1.97% compared to the surface-only sample (1.53%) and bulk-only sample (1.43%) in Figure 5c. Since the bulk-only sample comes from acid-washing of the surface-bulk-doped sample, the distribution of Ni^2+^ inside the bulk in both the bulk-only sample and the surface-bulk-doped sample should resemble each other. Similarly, the surface-only sample comes from the surface-replacement method of the pristine sample, and the distribution of Ni^2+^ on the surface in both the surface-only sample and the surface-bulk-doped sample should resemble each other as well, as observed in their respective XPS spectra and indicated by their STEM-EDS mapping results. Overall, the surface-bulk-doped sample has the Ni^2+^ distribution on the surface resembling the surface-only sample while the Ni^2+^ distribution in the bulk resembles the bulk-only sample, which explains the rationale for the doubled amount of Ni^2+^ doping in the surface-bulk-doped sample and will be addressed further in the mechanism section below.

Before digging into the photocatalysis mechanism, it is critical to assess all of the aforementioned evidence collectively to outline the overall structure and dopant distribution in the doped samples. The crystal structure, morphology, and surface properties after doping show no significant changes compared to the pristine brookite TiO_2_ nanoparticles. The surface-only samples prepared by post-treatment should contain only surface or subsurface defect sites replaced by Ni^2+^ ions, while bulk-only samples prepared by acid-washing should retain only the bulk-replaced Ni^2+^ ions, in which both have similar doping percentage. The surface-bulk-doped samples have evenly distributed Ni^2+^ ions from the surface to bulk with an almost doubled amount of doping concentration, which can account for both the surface and bulk doping effects. All of the evidence has provided a reliable basis for evaluating the photocatalytic performance and correlating the activity to the sole contributing factor in the mechanistic interpretation: the dopant ion-induced trapping sites, which act as mediators for promoting redox reactions. This will advance our fundamental knowledge of doping effects on semiconductor photocatalysts and potentially settle the long-standing debate of “To Dope or Not to Dope”^81^ and “Surface or Bulk.”^48^

Photocatalytic degradation of methylene blue serves as a model reaction to examine the effects of doping and distinguish the contributions of surface or bulk trapping sites generated by doping. The energy level of Ni^2+^ ions has a relatively moderate potential, allowing trapping sites to align closely with the Fermi level of brookite TiO_2_ (fully oxidized sample),^82,83^ which is beneficial for mediating the electron trapping and transfer from bulk excitation to surface reactions. Since the photogenerated holes are readily quenched by H_2_O to generate hydroxyl radicals (^•^OH), this aspect of the dynamics also relies on the electron dynamics controlled by the trapping sites.^44^ The overall rate-determining step for this photocatalyzed reaction heavily depends on the electron transfer rate at the semiconductor-adsorbate interface.^84^ Experimentally, the apparent reaction rate constant extracted from chemical kinetics can be used to examine this process over a relatively longer time scale. In heterogeneous photocatalytic degradation of organic compounds, the Langmuir–Hinshelwood (L-H) model and pseudo-order kinetic model are commonly applied to analyze the reaction rate and examine the mechanism.^85^ When the rate of degradation is significantly faster than the rate of adsorption, and the initial dye concentration is relatively low (<0.01 mol/L), the L-H model can be simplified into pseudo-first-order kinetics if the only variation is the dye concentration.^85^ In our photocatalytic system, the adsorption–desorption equilibrium was applied before any photocatalytic reaction (less than 10% of adsorption observed across all samples), and the MB dye concentration was about 3.1 × 10^–5^ mol/L, much smaller than the TiO_2_ photocatalyst concentration (about 0.0025 mol/L). Considering the relatively large concentration of dissolved oxygen (at 25 °C, about 2.6 × 10^–4^ mol/L and about 8.4 times larger compared to the MB) and excess water, the reactive oxygen species (superoxide and hydroxyl radicals from oxygen and water generated under photoexcitation) can be considered as excess reactants and should not affect the pseudo-first-order kinetics. Similar pseudo-first-order degradation of MB has been observed and also proposed in previous studies.^86–88^ We recognize that ultrafast transient absorption measurements are necessary to correlate the dynamics and observed kinetics more thoroughly, and we are in the process of pursuing this direction. However, as carefully evaluated in our proof-of-concept experiment, there is no competing process interfering with our interpretation of the trapping-site-induced mechanism, to the best of our knowledge, making it reasonable to correlate this kinetics analysis with the electron dynamics mentioned above.

As shown in Figure 6, both high-power mercury arc lamps and low-power white light (with a center wavelength of 575 nm) are used to evaluate the photocatalytic performance. Not surprisingly, the pristine brookite TiO_2_ exhibits a much higher reaction rate compared to the doped sample due to its rich intrinsic defects of Ti^3+^, which can act as natural trapping sites to mediate electron transfer. This phenomenon has been observed in several previous studies.^89–91^ More importantly, when the Ti^3+^ defect sites are depleted by Ni^2+^ ions, the reaction rates drop, displaying a distinguishable trend among surface-only, bulk-only, and surface-bulk-doped samples. As mentioned above, the doping percentage is observed to be quite similar for surface-only and bulk-only-doped samples, with the surface-bulk-doped samples having twice the amount, ensuring a similar distribution of Ni^2+^ across the lattice and the surface. Fundamentally, photoexcitation is a bulk phenomenon, and the electrons must migrate to the surface to mediate any reactions. Thus, the bulk-only or surface-only samples can capture these photogenerated electrons only locally at their doping sites, which limits their ability to further enhance the electron transfer chain. On the other hand, when the trapping sites are distributed more evenly from bulk to surface (as illustrated in Figure 7), the photogenerated electron/hole pairs not only can be separated temporarily with a much longer lifetime but also be spatially connected to the interfacial electron transfer. These continuous trapping-to-transfer sites could provide a more efficient pathway to utilize the photogenerated electrons and holes to produce reactive oxygen species (in this case, primarily superoxide radicals and hydroxyl radicals), which can target the MB molecules and ultimately lead to nontoxic products. As shown in Figure S11, a control experiment purged with Ar shows a lower degradation rate, confirming the contribution of superoxide radicals from dissolved oxygen reduction, while another control experiment with methanol as the hole scavenger shows a much lower degradation rate, confirming the contribution of hydroxyl radicals from water oxidation. It is in our current progress to quantify the amount of radicals generated over time and across different doped samples to further distinguish their contribution to the photocatalytic mechanism, but it is beyond the scope of this work. Overall, the surface-bulk-doped samples show a significant increase in their apparent reaction rate constants. It is worth mentioning that photocatalytic degradation may produce several intermediates that could potentially poison the catalyst’s surface or affect the kinetics, as observed in other studies;^92^ however, this is beyond the scope of this work.

**Figure 6:**
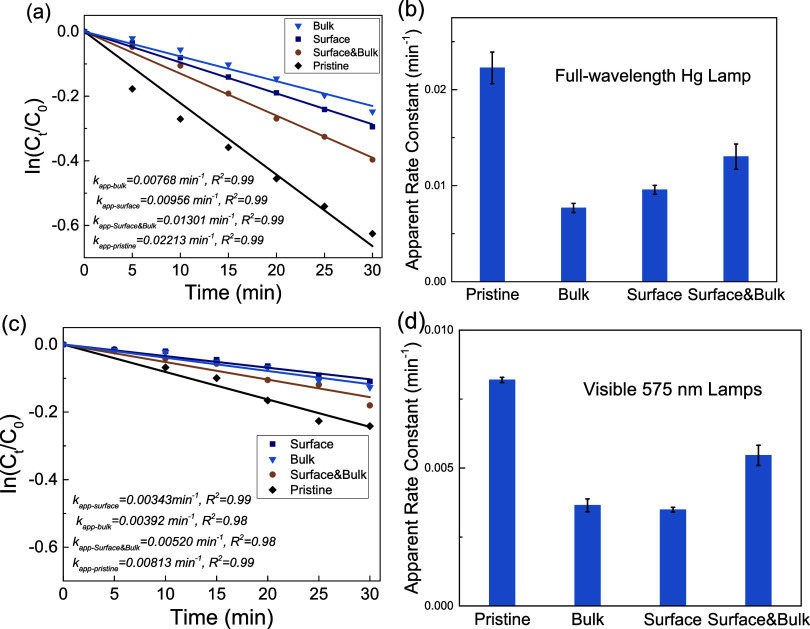
Kinetics analysis of photocatalytic performance with pristine brookite TiO_2_ nanoparticles, bulk-only Ni^2+^-doped, surface-only Ni^2+^-doped and surface-bulk Ni^2+^ doped TiO_2_ nanoparticles under (a, b) full-wavelength irradiation and (c, d) 575 nm visible-light irradiation. The photodegradation of methylene blue follows a pseudo-first-order reaction, and the linear fitting was plotted in parts (a, c) with solid lines. The apparent reaction rate constant was extracted from the linear fitting, and all error bars represent one standard deviation of the mean.

**Figure 7:**
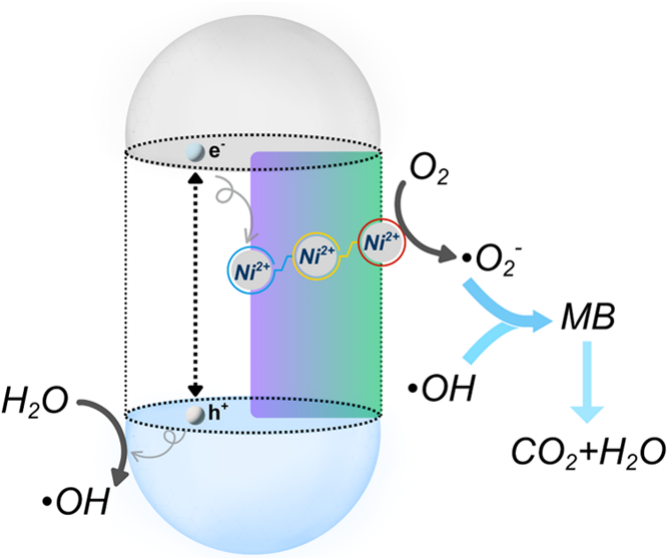
Proposed photocatalysis mechanism mediated by the continuous trapping sites from bulk to surface promotes the electron transfer at the semiconductor-adsorbate interface, which eventually enhances the reaction rate.

## Conclusions

In summary, we demonstrated a direct correlation between the doping position and subsequent photocatalytic performance in the Ni^2+^-doped brookite TiO_2_ nanoparticles while systematically and carefully examining the crystal structure, morphology, and surface composition to exclude other contributing factors to the performance. Ultimately, we propose that surface-only and bulk-only doping can only trap photogenerated electrons at their local sites, limiting their ability to mediate interfacial electron transfer. Continuous doping from bulk to surface is shown to be the most effective way to construct the trap-to-transfer chain, thereby enhancing reaction rates at the interface. This mechanistic insight can be applied to other transition-metal-ion-doped semiconductor photocatalysts, aiding in the design of more efficient photocatalytic systems.

## Supplementary Material



## Notes

Digital photographs of synthesized brookite TiO_2_ nanoparticles (Figure S1); K-M plot for band gap analysis measured from UV–vis DRS for pristine and doped samples (Figure S2); XRD patterns of synthesized pristine brookite TiO_2_ nanoparticles and standard PDF card (Figure S3); Rietveld refinement analysis of XRD patterns for pristine and doped samples (Figure S4); FTIR spectra of pristine and doped samples (Figure S5); SEM-EDS elemental mapping of Ti, O, and Ni in doped samples (Figures S6–S8); EPR spectrum of defects-depleted doped sample (Figure S9); high-resolution XPS spectra of C 1s in pristine and doped samples (Figure S10); control experiments with Ar-purged and methanol hole scavengers (Figure S11); XRD peak analysis for pristine and doped samples (Table S1) (PDF)
