# Supplementary material for: Surface or Bulk? Mechanistic Insights into Ni2+-Doped Brookite TiO2 Photocatalysts
Source: ACS Nanosci Au. 2025 Jul 30;5(4):324–36. doi: 10.1021/acsnanoscienceau.5c00087 (PMC12371590; doi:10.1021/acsnanoscienceau.5c00087)
Supplement: Supplementary file 1 [file ng5c00087_si_001.pdf]

Supporting Information for

Surface or Bulk? Mechanistic Insights into Ni^{2+}-doped Brookite TiO_2 Photocatalysts

Luke T. Coward,[1] Thu T. M. Chu,[2] Xiaotong Li,[2] Pin Lyu[1,*] and Oksana Love[1,*]

[1] Department of Chemistry and Biochemistry, University of North Carolina Asheville, 1 University Heights, Asheville, North Carolina 28804, United States.

[2] Department of Chemistry and Organic and Carbon Electronics Laboratories (ORaCEL), North Carolina State University, Raleigh, North Carolina 27695, United States.

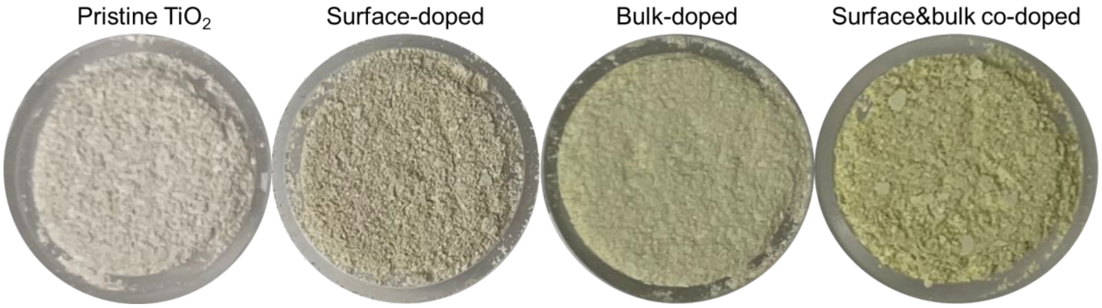

Figure S1. Digital photographs of synthesized brookite TiO_2 nanoparticles. From left to right: pristine, surface-only Ni^{2+}-doped, bulk-only Ni^{2+-}doped, and surface-bulk Ni^{2+} doped TiO_2 nanoparticles.

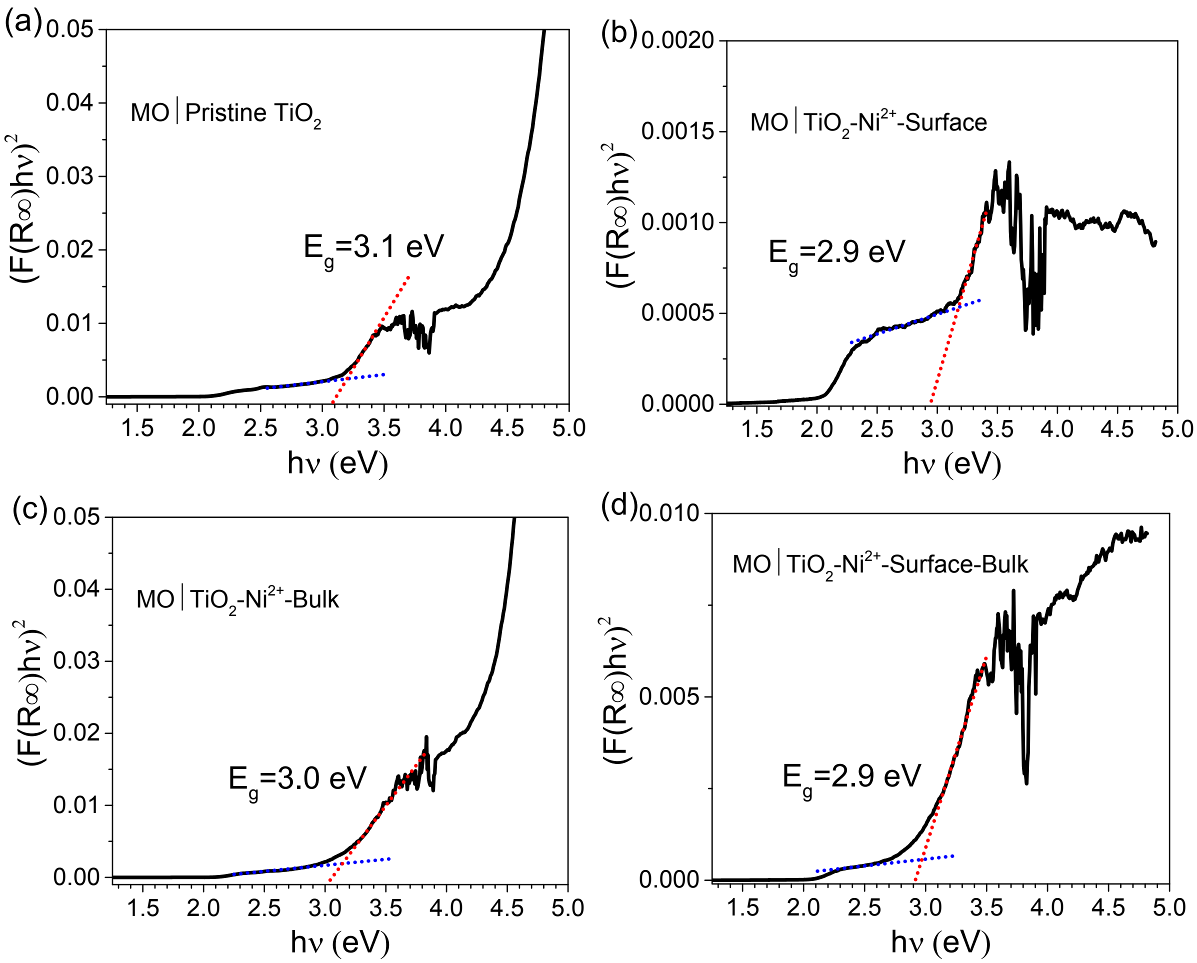

Figure S2. Transformed reflectance spectrum plots of MO|TiO_2 baseline approaches, where MO is methyl orange. (a) pristine brookite TiO_2 nanoparticles, (b) surface-only Ni^{2+}-doped, (c) bulk-only Ni^{2+}-doped and (d) surface-bulk Ni^{2+} doped brookite TiO_2 nanoparticles. The noise between 3.5 and 4.0 eV originates from the switching of different light sources, which does not affect our measurement below 3.5 eV.

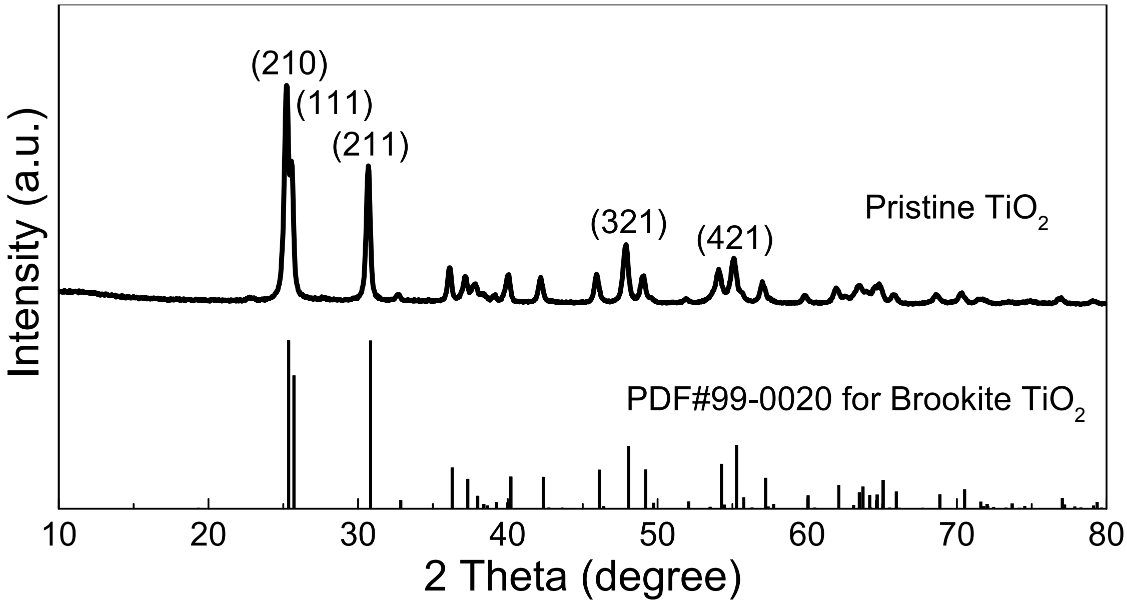

Figure S3. XRD patterns of synthesized pristine brookite TiO_2 nanoparticles and corresponding JCPDS standard card (PDF#99-0020). The five most intense peaks are labeled according to their crystal plane.

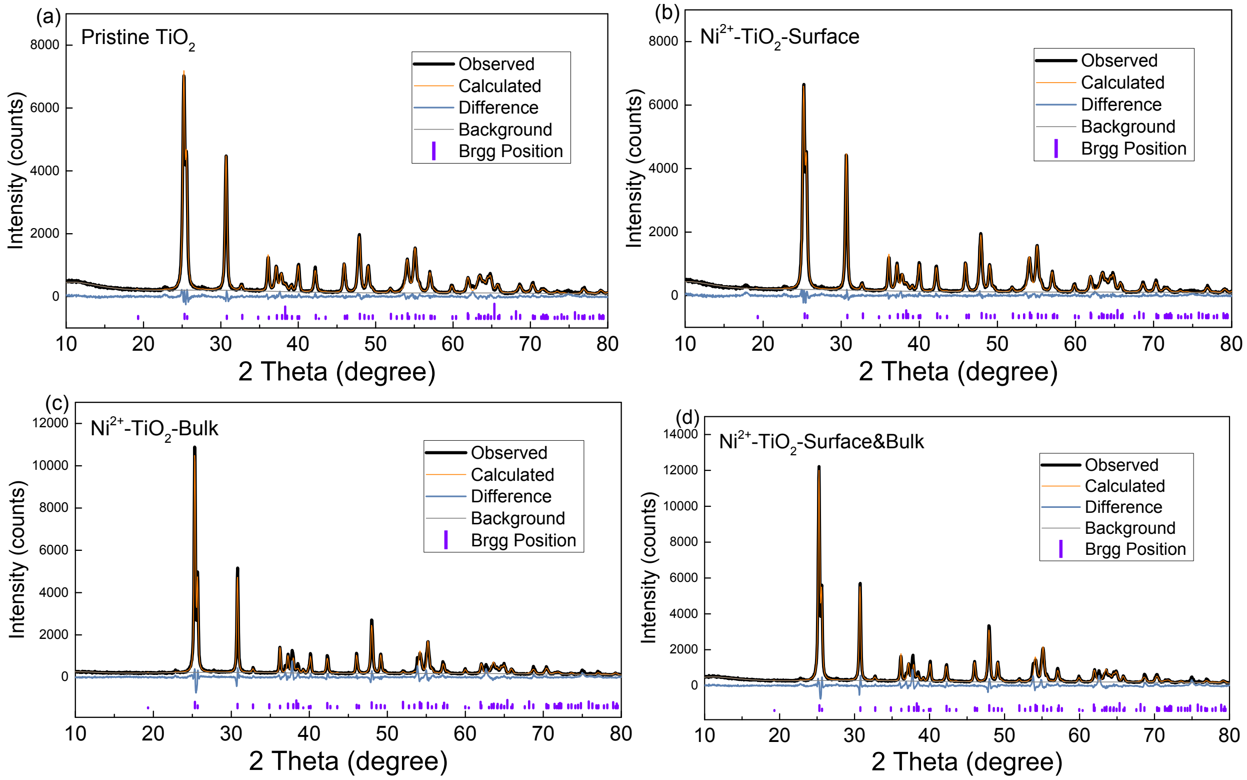

Figure S4. Rietveld refinement analysis of XRD patterns of (a) pristine brookite TiO_2 nanoparticles, (b) surface-only Ni^{2+}-doped, (c) bulk-only Ni^{2+}-doped and (d) surface-bulk Ni^{2+} doped TiO_2 nanoparticles. The refinement data were summarized in the table below.

Summary table of Rietveld refinement analysis of pristine and doped samples

Parameters	Pristine TiO_2	Ni^{2+}-TiO_2-Surface	Ni^{2+}-TiO_2-Bulk	Ni^{2+}-TiO_2-Surface&Bulk
Space Group	Orthorhombic, *P bca*			
a (Å)	9.1914	9.1894	9.1837	9.1885
b (Å)	5.4642	5.4612	5.4612	5.4656
c (Å)	5.1505	5.1491	5.1444	5.1471
R_p(%)	6.21	6.22	9.62	8.99
R_{wp} (%)	8.76	8.82	14.20	13.67
R_{exp} *(%)*	5.23	5.33	5.54	4.76
χ^2 (%)	2.81	2.74	6.57	8.25

Note: R_p for R-pattern and R_{wp} for R-weight pattern in fitting parameters to account for background subtracted and peak-only contributions. R_{exp} for expected R factor and the goodness of fit (χ^2) from the ratio between R_{wp} and R_{exp}.

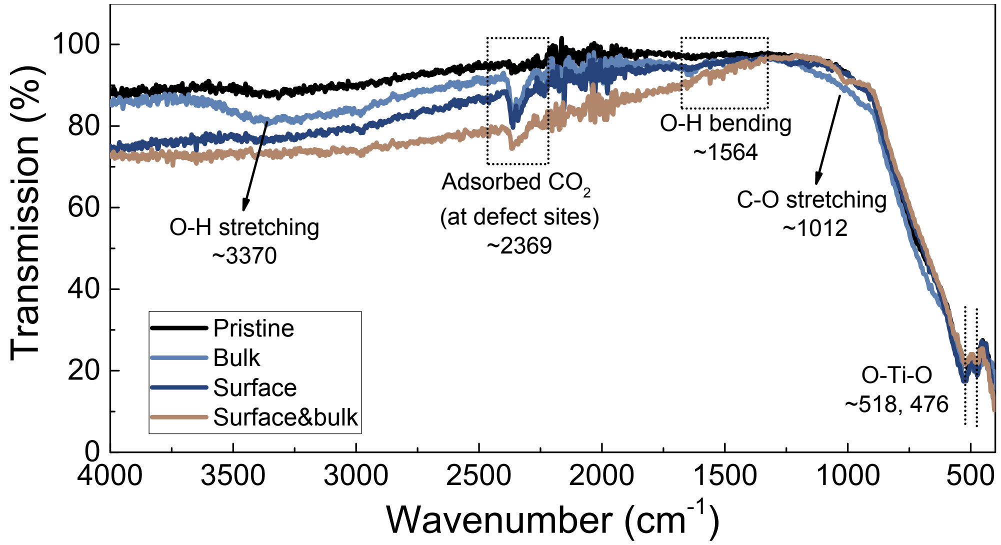

Figure S5. FTIR spectra of pristine, surface-only Ni^{2+}-doped, bulk-only Ni^{2+-}doped, and surface-bulk Ni^{2+} doped TiO_2 nanoparticles.

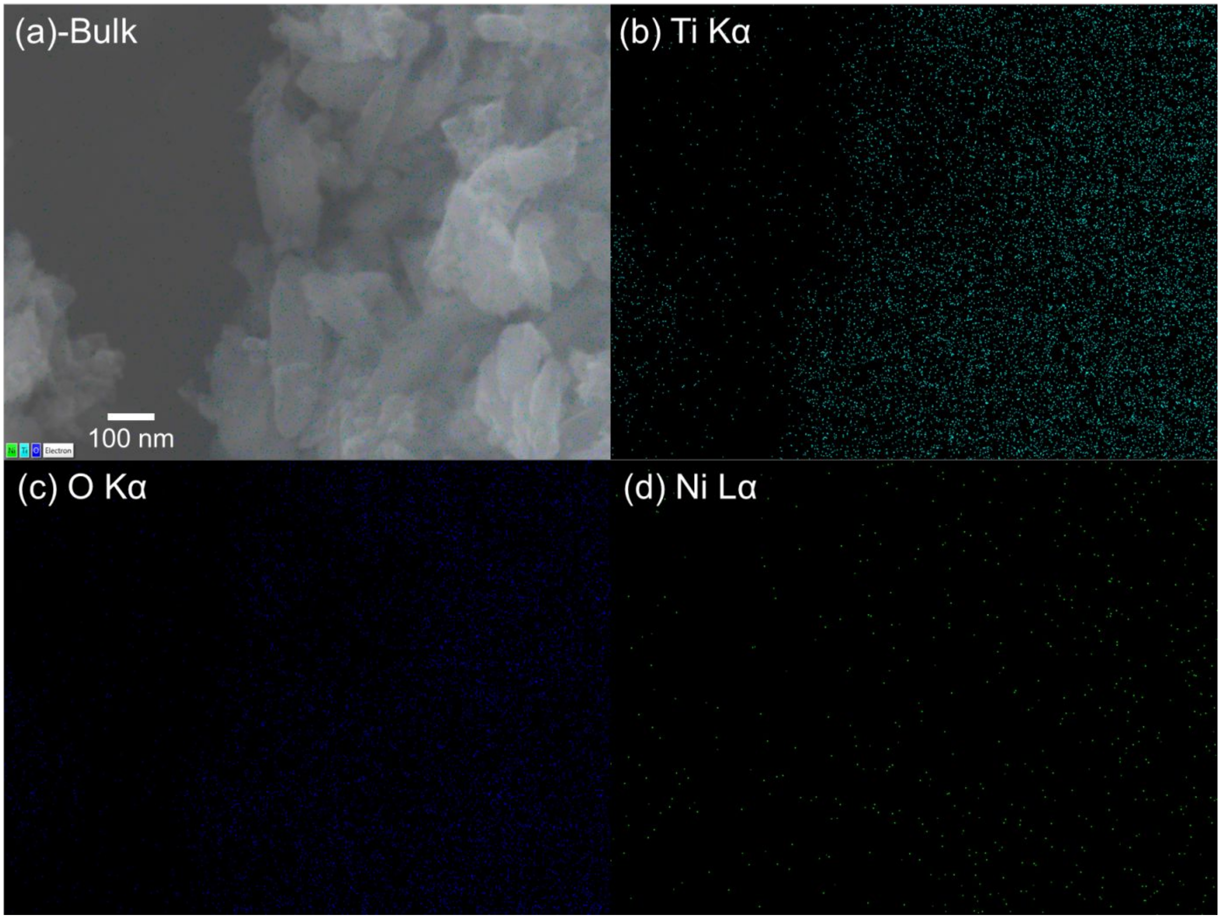

Figure S6. SEM-EDS elemental mapping of Ti, O, and Ni in the bulk-only Ni^{2+}-doped TiO_2 nanoparticles.

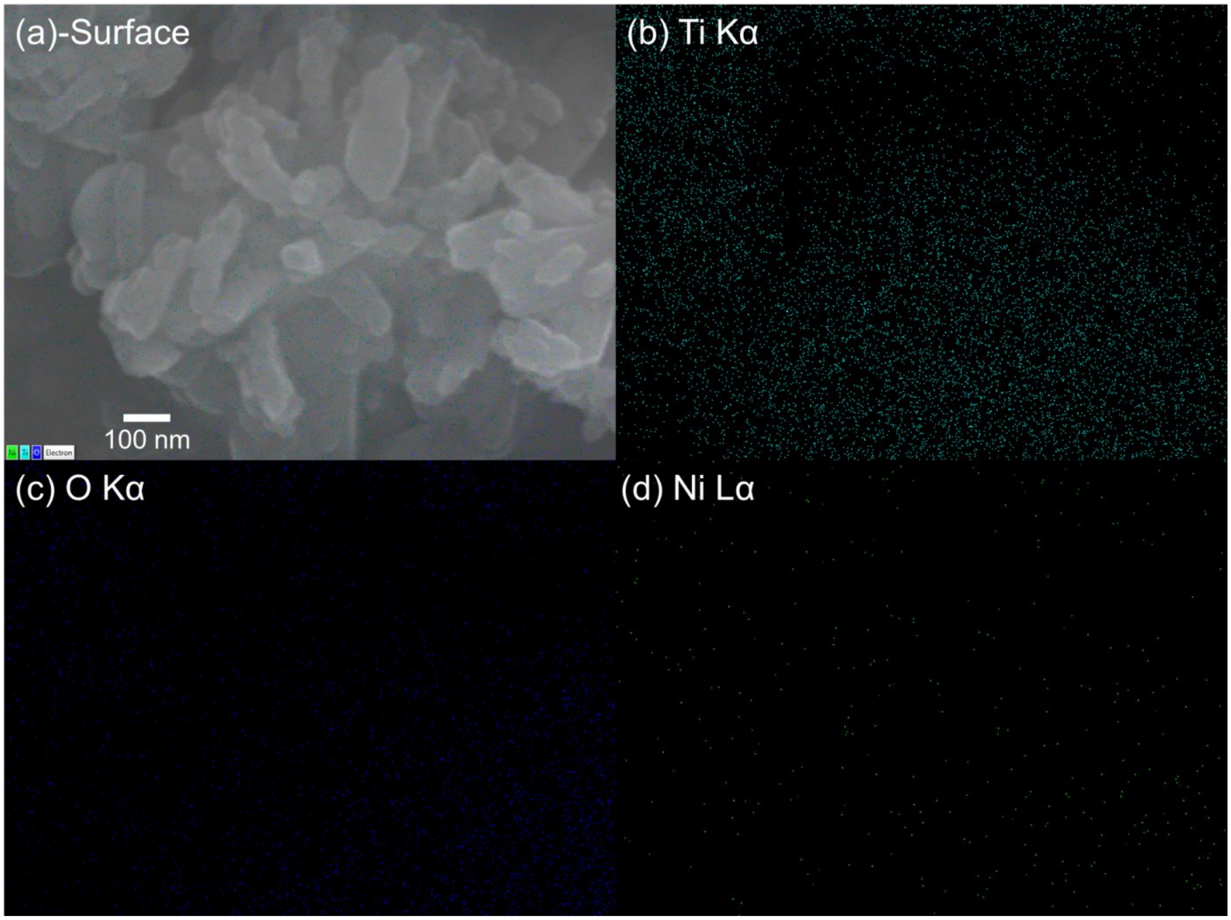

Figure S7. SEM-EDS elemental mapping of Ti, O, and Ni in the surface-only Ni^{2+}-doped TiO_2 nanoparticles.

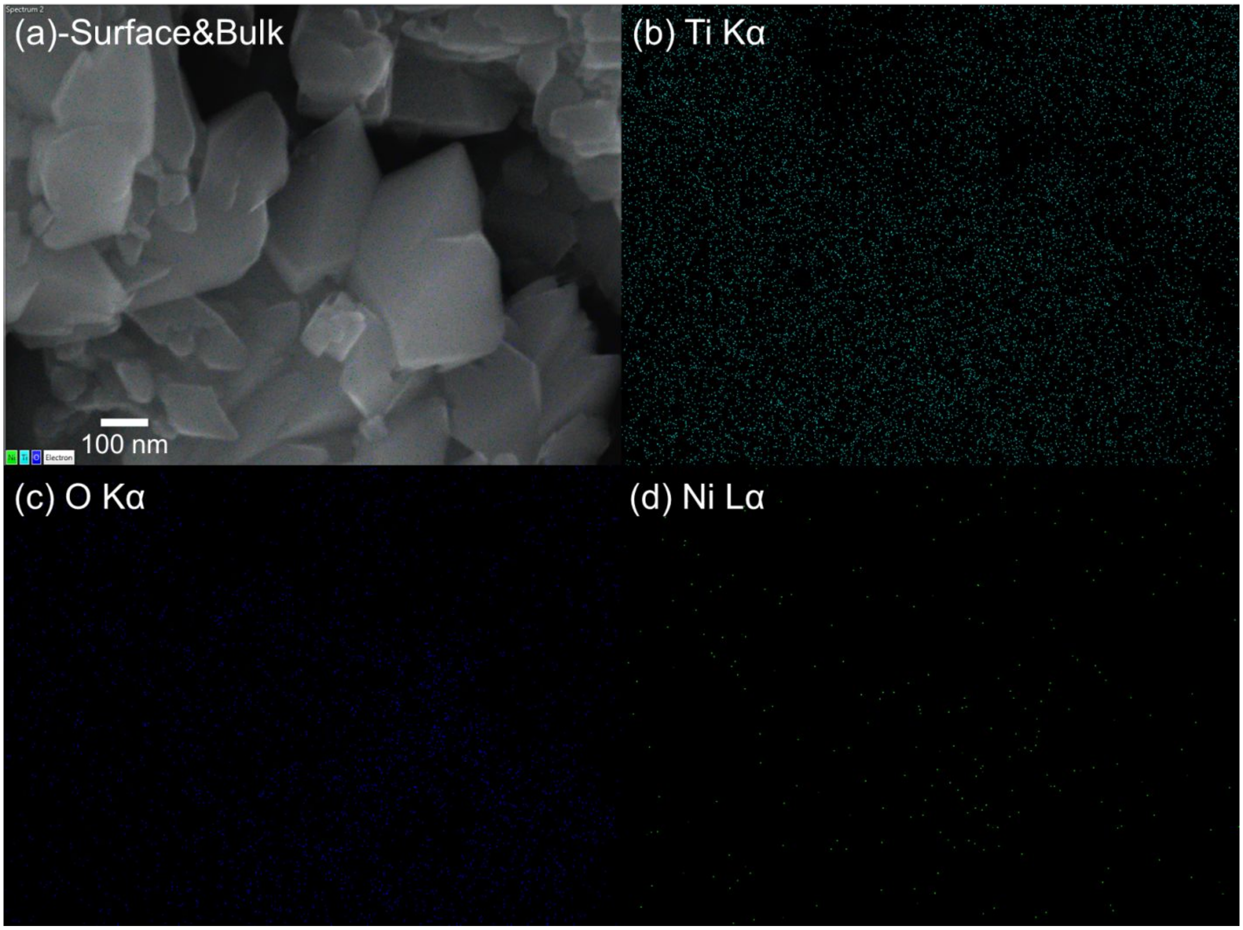

Figure S8. SEM-EDS elemental mapping of Ti, O, and Ni in the surface-bulk Ni^{2+} doped TiO_2 nanoparticles.

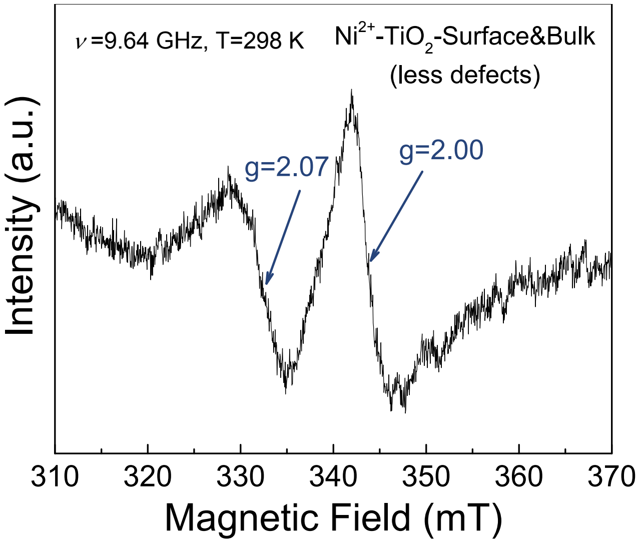

Figure S9. EPR spectrum of surface-bulk doped sample with less defects (stored under regular laboratory conditions without any protection for a year).

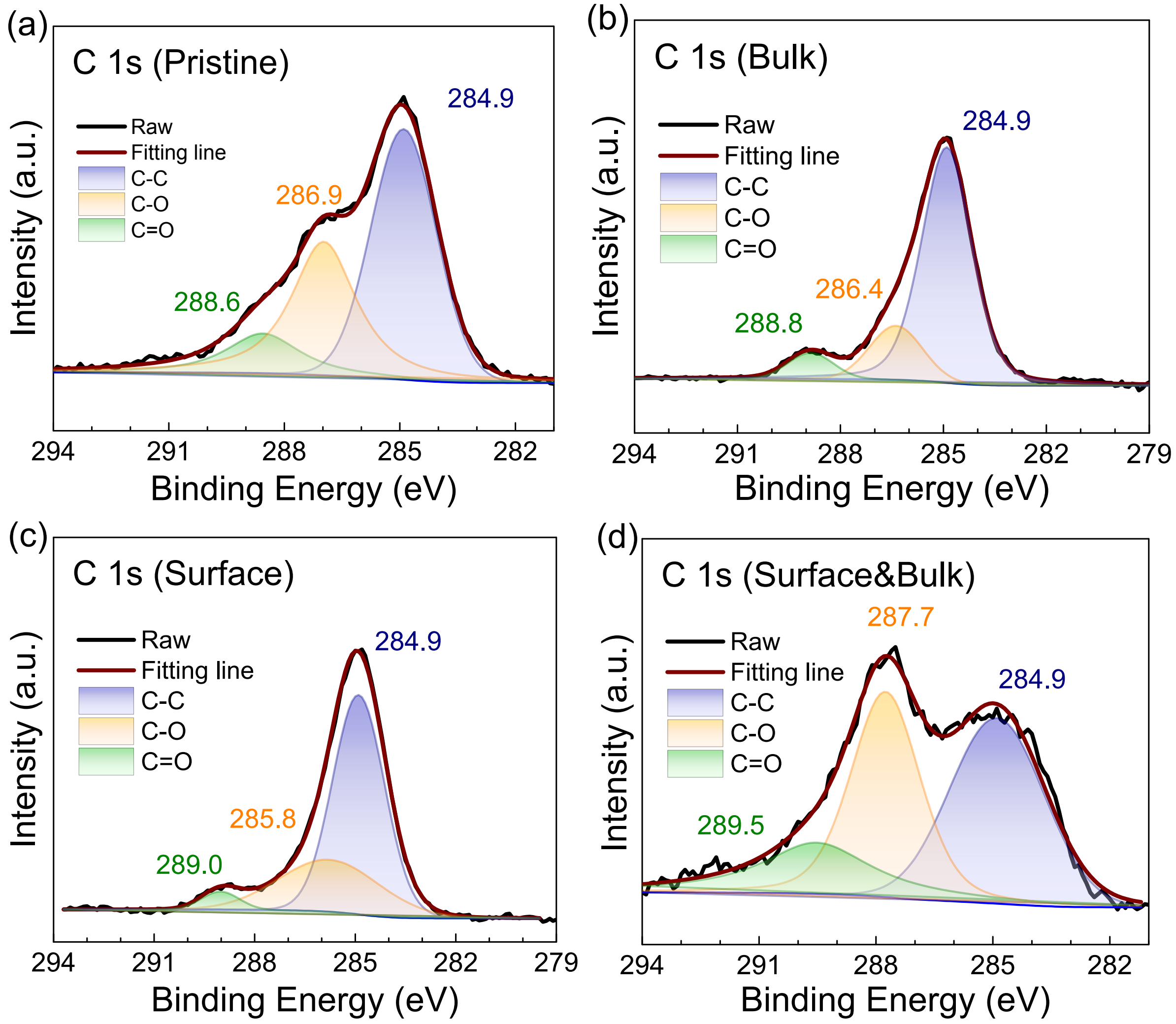

Figure S10. High-resolution XPS spectra of C 1s in (a) pristine brookite TiO_2 nanoparticles, (b) bulk-only doped, (c) surface-only doped, and (d) surface-bulk doped brookite TiO_2 nanoparticles. C-C represents single-bond species like sp^3 carbon in contamination carbon, C-O for single-bond species like surface hydroxyls, and C=O for double-bonds species like carboxylic acids.

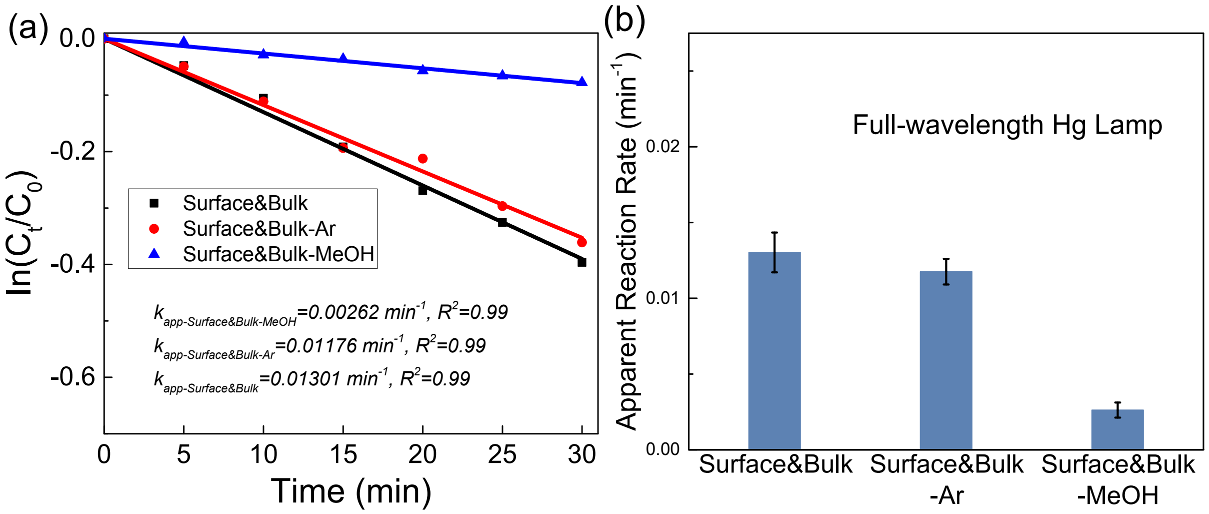

Figure S11. Control experiments of photocatalytic performance with surface-bulk Ni^{2+} doped TiO_2 nanoparticles with full-wavelength irradiation with Ar-purged and methanol hole scavengers. (a) Kinetics analysis and (b) Apparent reaction rate constant comparison. The apparent reaction rate constant was extracted from the linear fitting, and all error bars represent one standard deviation of the mean.

Table S1. XRD peak analysis for pristine and doped samples

Sample	Peak position (*2θ in º*)	FWHM (rad)	d-spacing (Å)	Peak position (*2θ in º*)	FWHM (rad)	d-spacing (Å)	Peak position (*2θ in º*)	FWHM (rad)	d-spacing (Å)	Crystallinity (%)
Pristine	25.127	0.315	3.54	25.496	0.202	3.49	30.636	0.359	2.91	77
Surface-only	25.174	0.267	3.53	25.523	0.203	3.49	30.666	0.309	2.91	81
Bulk-only	25.200	0.328	3.53	25.574	0.159	3.48	30.700	0.302	2.91	95
Surface &Bulk	25.154	0.305	3.54	25.523	0.180	3.49	30.652	0.209	2.91	95
